# Differential Effects of Pandemic (H1N1) 2009 on Remote and Indigenous Groups, Northern Territory, Australia, 2009

**DOI:** 10.3201/eid1709.101196

**Published:** 2011-09

**Authors:** James McCracken Trauer, Karen Louise Laurie, Joseph McDonnell, Anne Kelso, Peter Gregory Markey

**Affiliations:** Author affiliations: Centre for Disease Control, Tiwi, Northern Territory, Australia (J.M. Trauer, P.G. Markey);; World Health Organization Collaborating Centre for Reference and Research in Influenza, North Melbourne, Victoria, Australia (K.L. Laurie, A. Kelso);; Menzies School of Health Research, Tiwi (J. McDonnell)

**Keywords:** viruses, influenza A virus, H1N1, influenza, serology, health services, pandemic (H1N1) 2009, indigenous populations, high-risk populations, Australia, Northern Territory, research

## Abstract

Pandemic (H1N1) 2009 influenza spread through the Northern Territory, Australia, during June–August 2009. We performed 2 cross-sectional serologic surveys on specimens from Northern Territory residents, with 445 specimens obtained prepandemic and 1,689 specimens postpandemic. Antibody titers were determined by hemagglutination inhibition against reference virus A/California/7/2009 on serum samples collected opportunistically from outpatients. All specimens had data for patients’ gender, age, and address, with patients’ indigenous status determined for 94.1%. Protective immunity (titer >40) was present in 7.6% (95% confidence interval [CI] 5.2%–10.1%) of prepandemic specimens and 19.5% (95% CI 17.6%–21.4%) of postpandemic specimens, giving a population-standardized attack rate of 14.9% (95% CI 11.0%–18.9%). Prepandemic proportion of immune persons was greater with increasing age but did not differ by other demographic characteristics. Postpandemic proportion of immune persons was greater in younger groups and around double in indigenous persons. Postpandemic proportion immune was geographically heterogeneous, particularly among remote-living and indigenous groups.

## Abstract 2

TOC summary: Vaccination campaigns and public health responses should focus on high-risk groups.

Understanding the epidemiology of pandemic influenza is essential in directing public health responses, not only to the current pandemic, but also for recurrent waves of the same virus and future influenza pandemics. Knowledge of the distribution of protective immunity enables prediction of groups susceptible to reemergence of the virus and thus helps to improve efficacy of vaccine programs. Influenza has uneven effects across demographic and geographic groups, which may contribute to the increases in illness and death sometimes seen with subsequent waves (*1**,**2*). There is an emerging understanding of the effects of the outbreak of pandemic (H1N1) 2009 on indigenous populations, but little is known of the virus’s effect on remote and socio-economically disadvantaged groups.

Direct serologic measures of population immunity are useful in assessing the effect of pandemic influenza, as case or surveillance-based measures of incidence of infection are dependent on recognition of symptoms, use of health services, and subsequent testing (*3*). In remote and ethnically diverse populations, the differential effect of these factors may be particularly marked.

The Northern Territory (NT) is a jurisdiction unique for its large area of 1.35 million km^2^ (twice that of Texas) relative to its population of 225,000, of whom 30% are indigenous. The climate ranges from desert and semi-arid in central Australia to tropical in the northern “Top End” where the capital, Darwin, is located. There are also several smaller urban centers and many small, remote indigenous communities of 300–2,000 that may be >2 h flight from the nearest hospital. Indigenous Australians of the NT have considerably poorer health than the nonindigenous majority, with a life-expectancy gap of 15–20 years (*4*).

Following recognition of the pandemic (H1N1) 2009 virus in North America in April 2009, Australia experienced a single pandemic wave leading into the Southern Hemisphere winter (*5*). Despite enacting carefully prepared nationwide public health measures to delay viral entry and spread, widespread infection followed (*6**,**7*). Australia’s first case was reported on May 8, with the first case in the NT reported on June 2 and the first NT death occurring July 9 (*8*). Australia moved to the “protect” phase of its pandemic response on June 17 in an effort to limit illness and death from the virus (*9*), with notifications peaking nationwide and in the NT in July (*10*).

We undertook serosurveys using opportunistically collected outpatient serum specimens from persons across the NT to estimate levels of preexisting immunity and differential attack rates among demographic groups. Our study included a large proportion of remote-living persons, including Aboriginal and Torres Strait Islanders, enabling assessment of the differential effect of influenza upon these populations.

## Methods

### Specimens

Specimens were obtained from Western Diagnostic Pathology (Myaree, Western Australia, Australia), which provides outpatient pathology services covering most of the NT. Specimens were eligible for inclusion regardless of indication for testing, provided identifying information was complete and address was within the NT. We accepted only serum tubes with a residual volume >0.5 mL and obtained specimens before routine discarding. Baseline specimens were selected during January–May and all postpandemic specimens from September 2009.

### Background Information

Data obtained for each specimen consisted of date of collection, patient’s age in years at collection, gender, suburb/community of address, and a unique study identifier. Identifying data (name and date of birth) were transferred directly from the laboratory to the Information Services Division of the NT Department of Health and Families for computer-matching to indigenous status. This was successful in 94.1% of cases, and the data were transferred to the investigators linked to the study identifier. Of those cases with a successful match, 59.7% of patients were neither indigenous nor Torres Strait Islander, 39.7% were Aboriginal, 0.1% were Torres Strait Islander, and 0.6% were both Aboriginal and Torres Strait Islander. The suburb of patient’s address for each specimen was linked to 2006 Statistical Local Area (SLA), the Australian Bureau of Statistics’ general purpose base spatial unit, with 82 of 96 NT SLAs represented (*11*).

**Figure 1:**
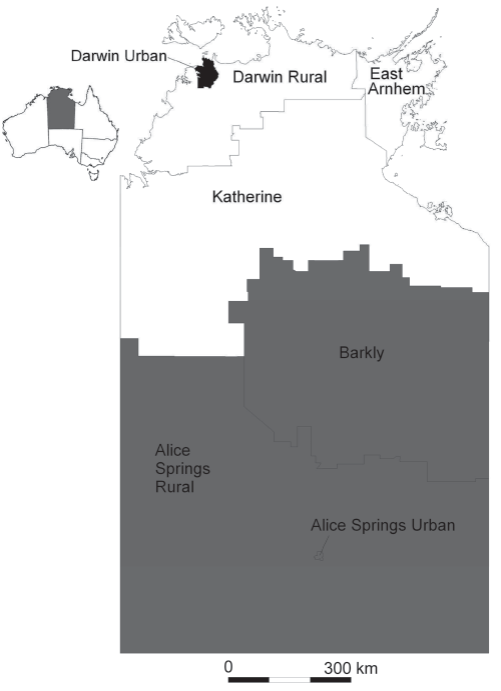
Health districts, by study region, in a study of differential effects of pandemic (H1N1) 2009 on remote and indigenous groups, Northern Territory, Australia, September 2009. Black, Urban Darwin; white, Rural Top End; gray, Central Australia. Inset: Location of the Northern Territory in Australia.

After testing, a small number of specimens were redistributed by region, following manual review of suburb of address linkage to SLA. The SLA code was also linked to the 11 statistical subdivisions and 7 health districts in the NT. Three study regions were defined, displayed in Figure 1, consisting of Urban Darwin; Rural Top End (Darwin Rural, East Arnhem, and Katherine districts); and Central Australia (Alice Springs Urban, Alice Springs Rural, and Barkly districts). SLA codes were then linked to the Australian Bureau of Statistics’ Socio-Economic Indexes for Area (SEIFA) (*12*). These measures use information from census data relating to material and social resources and ability to participate in society to obtain a broad level of relative socioeconomic status for each SLA. For calculation of attack rates by quintile, the SEIFA index of relative disadvantage was used, while for regression analysis, the SEIFA index of relative advantage and disadvantage was preferred, as this index does not incorporate indigenous status.

### Laboratory Methods

Antibody responses to pandemic (H1N1) 2009 influenza were assessed at the World Health Organization Collaborating Centre for Reference and Research on Influenza in North Melbourne, Victoria, Australia. Reactivity of serum against pandemic (H1N1) 2009 influenza was measured in 2140 serum samples by using hemagglutination inhibition (HI). Egg-grown A/California/7/2009 virus was purified by sucrose gradient, concentrated, and inactivated with β-propiolactone to create an influenza zonal pool preparation (a gift from CSL Ltd., Parkville, Victoria, Australia). Serum samples were pretreated with 1:4 vol/vol receptor-destroying enzyme II (Deka Seiken Co. Ltd., Tokyo, Japan) at 37°C for 16 h, then enzyme was inactivated by the addition of an equal volume of 54.4 mmol/L trisodium citrate (Ajax Chemicals, Taren Point, New South Wales, Australia) and incubated at 56°C for 30 min. A total of 25 μL (4 hemagglutinin units) influenza zonal pool preparation A/California/7/2009 virus or 25 μL phosphate-buffered saline (“no virus” control) was incubated at room temperature with an equal volume of receptor-destroying enzyme–treated serum. Serum specimens were titrated in 2-fold dilutions in phosphate-buffered saline from 1:10 to 1:1280. After a 1-h incubation, 25 μL of 1% vol/vol turkey erythrocytes wase added to each well. HI was read after 30 min. Any samples that bound the erythrocytes in the absence of virus were adsorbed with erythrocytes for 1 hour and reassayed. Six samples bound erythrocytes in the absence of virus and were excluded from analysis. Titers were expressed as the reciprocal of the highest dilution of serum at which hemagglutination was prevented.

A panel of control and serum samples were run in addition to the test serum samples for all assays. The control panel comprised paired ferret serum samples pre- and postinfection with pandemic (H1N1) 2009; seasonal influeza A (H1N1), A (H3N2), or B viruses; and paired human plasma and serum samples from donors, collected before April 2009 or after known infection with pandemic (H1N1) 2009 or vaccination with the Australian monovalent pandemic (H1N1) 2009 vaccine.

### Study Population

We aimed to estimate the proportion of persons with serologic immunity in each of 12 groups in the post-pandemic sample, consisting of 4 age groups (<14, 15–34, 35–54, and >55 y) within each of the 3 study regions described. In the postpandemic group, we calculated a required sample size of 195 specimens per group (for a total of 2,340 specimens) on the basis of an estimate of 15% immunity with a 95% confidence interval (CI) of 10%–20%.

In the baseline group, we aimed to provide an age-specific, NT-wide estimate of preexisting immunity and calculated a single sample size for each of the same 4 age groups described. We did not stratify by region and assumed increasing prepandemic immunity with age (2% in those <14 y, 5% in those 15–54 y, and 15% in those >55 y).

Samples were chosen at random from each stratum and checked for representativeness of the NT population by gender and region before testing. Data on indigenous status were obtained from Information Services only after final selection of specimens.

### Analytic Methods

For all analyses, immunity was defined as an HI titer ≥40, consistent with published data (*13*) and the observation that titers of this order develop in 90% of persons <21 days of illness (*14*). Attack rates were calculated as the difference in proportion immune between the September category and the total baseline group, except for age-specific attack rates where the age-specific baseline proportion was used as the reference. All attack rate calculations were population standardized, with weights calculated separately for pre- and postpandemic samples, based on the demographic characteristics of the 2009 NT population by age-group, indigenous status, and study region. Regression models and proportions immune are displayed unweighted. Statistical analysis was performed with Stata version 11.0 (StataCorp LP, College Station, TX, USA).

### Ethical Approval

We obtained ethical approval from the Menzies School of Health Research Human Research Ethics Committee and the Central Australia Human Research Ethics Committee. We continued to liaise with the Aboriginal and Torres Strait Islander subcommittees of both ethics committees throughout the study.

## Results

### Baseline Immunity

A total of 445 specimens taken January 10–May 29 were selected from 10,575 available serum tubes (Table 1). Within each age group sampled, the baseline sample was representative of the 2009 NT population (*15*) by gender, indigenous status, and study region, except that higher proportions of indigenous Australians were seen in the 2 older age brackets. There was no tendency toward an increase in the proportion of specimens with protective immunity over the 5 months from which baseline specimens were taken (p = 0.79, by χ^2^ test for trend).

**Table 1:** Demographic characteristics of patients in a study of differential effects of pandemic (H1N1) 2009 on remote and indigenous groups, Northern Territory, Australia, 2009

Characteristic	No. patients	Female, %	Indigenous, %
Baseline, age, y			
<14	37	54.1	51.4
15–34	91	65.9	43.5
35–54	92	54.4	33.7
>55	225	44.9	30.3
Total	445	51.9	35.5
September, Urban Darwin, age, y			
<14	60	53.3	14.0
15–34	194	62.9	13.5
35–54	202	55.5	9.6
>55	209	48.3	6.7
Total	665	55.2	10.2
September, Rural Top End, age, y			
<14	25	36.0	44.0
15–34	190	60.5	71.4
35–54	183	47.5	60.8
>55	190	46.8	42.5
Total	588	51.0	57.8
September, Central Australia, age, y			
<14	13	46.2	61.5
15–34	84	57.1	82.7
35–54	189	63.5	63.2
>55	150	51.3	54.9
Total	436	57.6	64.1

A total of 34 of 445 baseline specimens (7.6%, 95% CI 5.2%–10.1%) had HI titers >40. Multivariate logistic regression revealed no difference in baseline immunity by gender, indigenous status, study region, or index of socioeconomic disadvantage (p>0.05), with increasing age in years the only significant independent predictor of prepandemic immunity (p = 0.003). Although not statistically significant on the regression model, the proportion of specimens with titers >40 appeared higher in Central Australia (14.0%) than Urban Darwin and Rural Top End (5.6%). Immunity was nevertheless evenly spread geographically within these regions.

### Postpandemic Immunity

**Figure 2:**
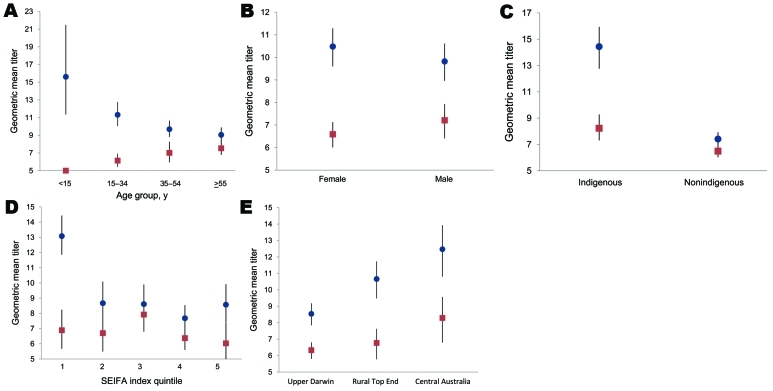
Unadjusted geometric mean antibody titers by age group (A), sex (B), indigenous status (C), SEIFA index (D), and study region (E) in a study of differential effects of pandemic (H1N1) 2009 on remote and indigenous groups, Northern Territory, Australia, September 2009. Red, prepandemic titer; blue, postpandemic titer. Bars indicate range. SEIFA, Australian Bureau of Statistics’ Socio-Economic Indexs for Area.

**Figure 3:**
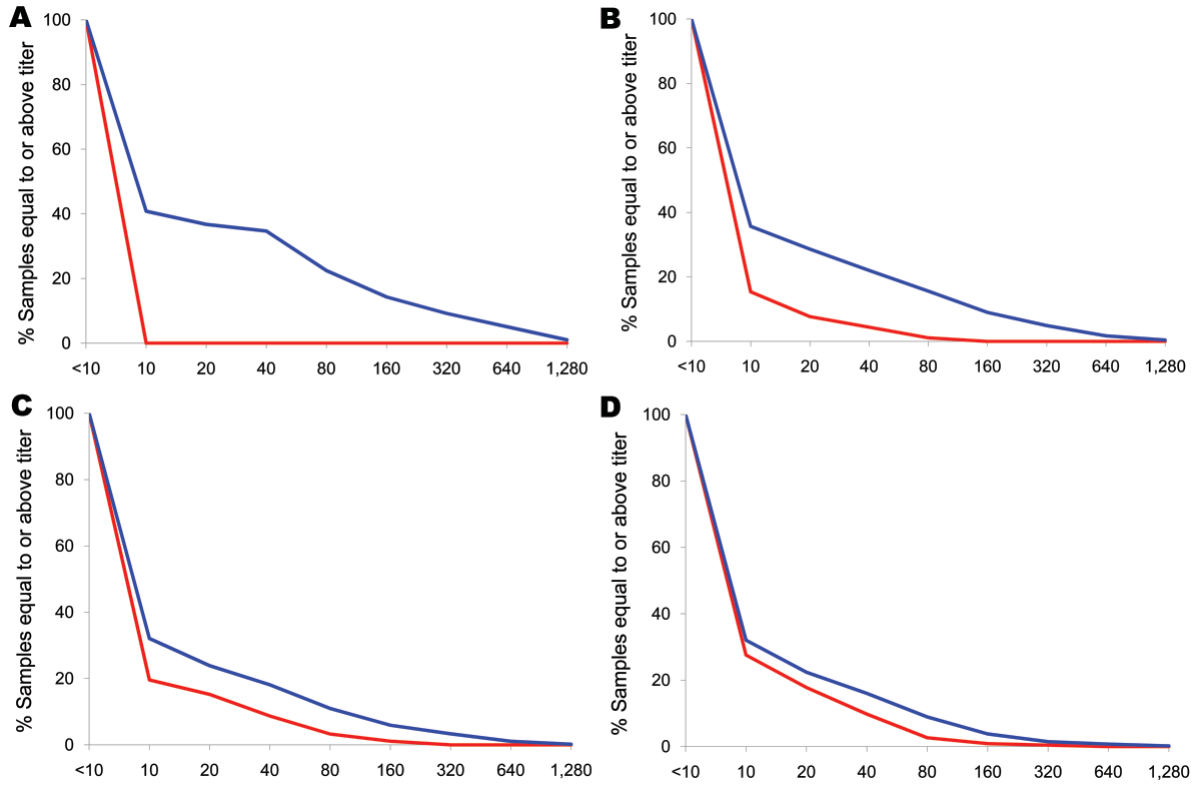
Reverse cumulative distributions by age group in a study of differential effects of pandemic (H1N1) 2009 on remote and indigenous groups, Northern Territory, Australia, September 2009, showing percentage of population with titer at or above each value. A) <15 years of age; B) 15–34 years of age; C) 35–54 years of age; D) >55 years of age. Red, prepandemic titer; blue, postpandemic titer.

A total of 1,689 specimens collected September 3–30, 2009, were selected from 3,228 available. Because of insufficient numbers of specimens, the required sample size was not achieved in 5 of 12 postpandemic groups. The September samples were representative of the 2009 NT population by gender but again included higher proportions of specimens from indigenous Australians in the older age brackets. An HI titer >40 was seen in 329 specimens (19.5%, 95% CI 17.6%–21.4%), with proportions by study group shown in Table 2, geometric mean titers in Figure 2, and reverse cumulative distributions in Figure 3. There was a nonsignificant trend toward a decreasing proportion of specimens with protective immunity over the 5 weeks from which the September specimens were taken (p = 0.20, by χ^2^ test for trend).

**Table 2:** Specimens with titers >40 in a study of differential effects of pandemic (H1N1) 2009 on remote and indigenous groups, Northern Territory, Australia, September 2009*

Population group	No. positive/ no. tested	% Titers >40 (95% CI)
Baseline, age, y		
<14	0/37	0
15–34	4/91	4.4 (0.1–8.6)
35–54	8/92	8.7 (2.9–14.5)
>55	22/225	9.8 (5.9–13.7)
Urban Darwin, age, y		
<14	22/60	36.7 (24.3–49.0)
15–34	34/194	17.5 (12.2–22.9)
35–54	23/202	11.4 (7.0–15.8)
>55	20/209	9.6 (5.6–13.6)
Rural Top End, age, y		
<14	5/25	20.0 (4.0–36.0)
15–34	46/190	24.2 (18.1–30.3)
35–54	31/183	16.9 (11.5–22.4)
>55	35/190	18.4 (12.9–24.0)
Central Australia, age, y		
<14	7/13	53.9 (25.6–82.1)
15–34	23/84	27.4 (17.8–37.0)
35–54	50/189	26.5 (20.1–32.8)
>55	33/150	22.0 (15.3–28.7)

*CI, confidence interval.

Table 3 shows the results of multivariate logistic regression analysis for the 1,592 postpandemic specimens for which the indigenous status of patients was known. No association was detected between immunity and gender, socioeconomic status, or study region. However, younger age and indigenous status were independently associated with immunity. A measure of remoteness was examined as a possible exposure variable, but colinearity with region meant that it was not a useful predictor variable and therefore was not included in regression analysis (*16*).

**Table 3:** Multivariate logistic regression for exposures associated with titer >40 in a study of differential effects of pandemic (H1N1) 2009 on remote and indigenous groups, Northern Territory, Australia, September 2009

Characteristic	Odds ratio (95% confidence interval)	p value
Female sex	1.06 (0.82–1.37)	0.65
Aboriginal and Torres Strait Islander	2.32 (1.63–3.31)	<0.001
Age, y		<0.001
>55	Reference	
35–54	1.05 (0.76–1.45)	
15–34	1.28 (0.91–1.79)	
<14	2.98 (1.80–4.92)	
Region		0.05
Urban Darwin	Reference	
Rural Top End	0.83 (0.56–1.23)	
Central Australia	1.23 (0.80–1.90)	
Socioeconomic quintile*		0.43
5 (least disadvantaged)	Reference	
4	0.91 (0.55–1.51)	
3	1.16 (0.73–1.86)	
2	1.41 (0.84–2.36)	
1 (most disadvantaged)	1.21 (0.70–2.12)	

*Australian Bureau of Statistics’ Socio-Economic Indexes for Area index of relative socioeconomic advantage and disadvantage.

**Figure 4:**
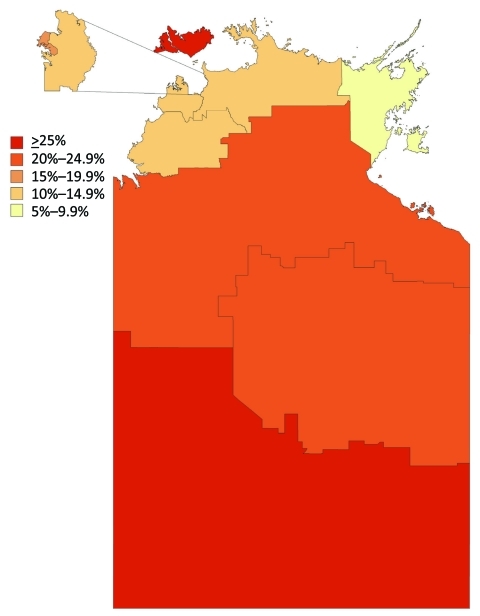
Postpandemic proportion immune by statistical subdivision in a study of differential effects of pandemic (H1N1) 2009 on remote and indigenous groups, Northern Territory, Australia, September 2009. Inset represents Urban Darwin.

**Figure 5:**
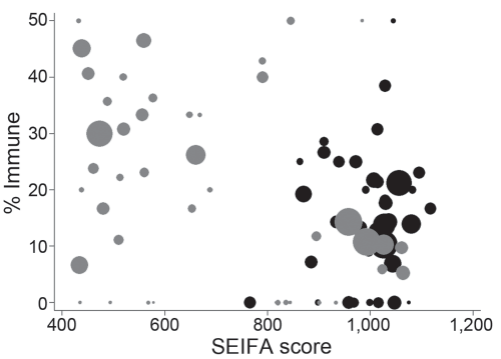
Postpandemic proportion of Statistical Local Area (SLA) demonstrating titers >40 by Socio-economic Index for Area (SEIFA) of relative socioeconomic disadvantage. Gray circles, Urban Darwin; black circles, Rural Top End and Central Australia. Circle size proportional to number of specimens in group. Lower score indicates greater degree of relative socioeconomic disadvantage. One SLA containing 1 observation with a proportion immune of 100% is not displayed.

The proportion immune in September was geographically heterogeneous across the 3 study regions (p<0.001, by χ^2^ test). The same pattern was seen for Statistical Subdivisions (p<0.001, by χ^2^ test), with proportionate immunity ranging from 7.5% to 42.9%, as illustrated in Figure 4. The picture of heterogeneity was also seen for the indigenous population considered separately. However, the prevalence of postpandemic immunity was more homogeneous for the nonindigenous population considered by either geographic classification and for urban Darwin considered separately. Figure 5 demonstrates that while postpandemic levels of immunity were relatively homogeneous by SLA in less disadvantaged, generally urban areas, comparatively disadvantaged areas had more variable levels of immunity.

### Attack Rates

As shown in Table 4, attack rates by age group were markedly higher in younger groups, reaching approximately 1 in 3 among children <14 years of age. Indigenous Australians were also disproportionately affected, with attack rates of ≈1 in 4, which were 1.85-fold higher than those seen in nonindigenous Australians. No differences in attack rates were seen by gender, region, or socioeconomic quintile. Given these attack rates, we estimate that 15,600 (95% CI 10,900–20,300) of 67,820 indigenous and 19,500 (95% CI 12,700–26,300) of 157,028 nonindigenous persons in the NT acquired pandemic influenza during May–September 2009.

**Table 4:** Attack rates standardized to Northern Territory population, by age group, indigenous status, and geographic region, in a study of differential effects of pandemic (H1N1) 2009 on remote and indigenous groups, Northern Territory, Australia, September 2009

Demographic characteristics	Adjusted attack rate, % (95% Confidence interval)
Overall	14.9 (11.0–18.9)
Sex	
F	15.4 (10.7–20.0)
M	14.4 (9.1–19.7)
Aboriginal and Torres Strait Islander	22.9 (16.0–29.9)
Nonindigenous	12.4 (8.1–16.8)
Age, y	
<14	36.0 (25.5–46.4)
15–34	15.3 (9.8–20.9)
35–54	4.3 (−3.2 to 11.8)
>55	3.5 (−1.2 to 8.2)
Geographic region	
Urban Darwin	12.8 (8.4–17.2)
Rural Top End	14.2 (8.0–20.4)
Central Australia	21.4 (12.8–30.1)
Socioeconomic quintile*	
5 (least disadvantaged)	13.6 (7.5–19.8)
4	10.0 (4.3–15.7)
3	14.6 (7.5–26.8)
2	24.0 (14.6–33.5)
1 (most disadvantaged)	13.8 (6.9–20.6)

*Australian Bureau of Statistics’ Socio-Economic Indexes for Area index of relative socioeconomic advantage and disadvantage.

## Discussion

Our study is an outpatient-based serologic survey of the impact of pandemic influenza over a large geographic region. Because of our broad sampling base, we have been able to estimate attack rates across the NT population and to assess the differential impact of the virus on the indigenous population. We calculated a population attack rate of ≈15% but found marked differences in patterns of exposure by indigenous status, geographic location, and age. Younger age groups and indigenous Australians were disproportionately affected, with striking geographic variations seen.

Baseline immunity could be overestimated if undetected virus circulation was occurring during our prepandemic period. We believe this is unlikely, as there was no trend toward increasing immunity in samples taken at a later date, no child had a baseline titer >10, and the first confirmed case was not detected in the NT until May 29 (*17*). Similarly, our September sample could have underestimated true postpandemic immunity caused by ongoing infection during this month. However, emergency department presentations of influenza-like illness had returned to baseline by this time, and there were few laboratory-confirmed cases during this period. Similarly, no increase in immunity was observed during September in our study. Because the national pandemic vaccination program in Australia commenced in NT on September 30, testing of specimens before this date would be unaffected by antibodies produced by vaccination (*18*).

Although we attempted to ensure that our sample was demographically representative of the NT population, the prevalence of risk factors for influenza infection may be different in our sample from that of the general population. In particular, chronic disease and pregnancy may have been overrepresented among patients presenting for outpatient pathologic analysis. However, because clinical data, including indication for testing, were not available, the strength of this possible effect cannot be assessed.

We found a prevalence of preexisting immunity of 3.6% in those born after 1980 and of 0% in children. In those born before 1950, the level of preexisting immunity was 13.7%, which is lower than data from North America (*19**,**20*). This may reflect regional differences or be the result of the 1976 mass-vaccination campaign against swine-origin H1N1 virus because seasonal influenza vaccination does not produce protective titers against the pandemic (H1N1) 2009 virus. Despite this finding, serologic data from a population in China with low seasonal vaccine coverage found lower levels of preexisting immunity (*21*), although comparable levels of preexisting immunity were seen in Singapore (*22*).

Our findings of a postpandemic proportion immune rate of 19.5%, attack rate of 14.9%, and the association with younger age are consistent with other published data (*14**,**22*), although the difference in post-pandemic immunity in the Australian indigenous population has not been reported. Our overall attack rate was notably higher than the estimated clinical attack rate of 7.2% extrapolated from surveillance data (*23*). Moreover, the incidence rate ratio between indigenous and nonindigenous populations based on the number of laboratory-confirmed cases was 4.9, notably greater than our 2-fold ratio. However, the ratio in serologic attack rates between Central Australia and the Top End was ≈1.5 and consistent with the ratio from laboratory-confirmed cases (*23*). Data from notifications and hospitalizations in the Top End indicate that the prevalence of risk factors in patients admitted with pandemic influenza was similar between indigenous and nonindigenous patients (*24*), suggesting that the increased frequency of admissions in indigenous persons was because of the greater prevalence of risk factors for severe disease.

Australian indigenous populations have more respiratory infections than nonindigenous groups (*25*), and the higher pandemic attack rate in this group is also consistent with their overrepresentation in admissions to intensive care units (*26*). North American indigenous persons also have a greatly increased risk for hospitalization and death from pandemic (H1N1) 2009 influenza compared with their nonindigenous counterparts, particularly at extremes of age (*27*). However, Australia has among the greatest differences in rates of hospitalization and mortality between indigenous and nonindigenous populations in the Americas and Pacific regions (*28*). For this reason, the NT Centre for Disease Control has identified this group as a particular focus of the univalent pandemic influenza vaccination program, achieving coverage of 24% overall and 41% in the indigenous population.

We used the Australian Bureau of Statistics SEIFA index as our measure of relative socioeconomic disadvantage (*12*). By this measure, the most disadvantaged quintile appeared to have higher rates of infection. However, this finding was not borne out by multivariate analysis, suggesting confounding by other variables, particularly indigenous status and remoteness, which are highly correlated in NT. Moreover, accurate estimates of socioeconomic disadvantage are notoriously difficult to attain (*29*) and, when measured by area, are at best at an average level of deprivation.

We observed marked differences in the postpandemic proportion immune between Statistical Subdivisions, with the degree of heterogeneity being particularly prominent among indigenous and remote populations. This variability in influenza infections has been noted from surveillance data (*30*) and serologic survey data (*14*). Although many Aboriginal communities in the NT are remote and isolated, a large proportion of persons from remote communities demonstrate intercommunity mobility (*31*). The Aboriginal population of the NT is known to have high rates of chronic diseases, including conditions identified as increasing susceptibility to influenza (*4**,**32*), such as poor housing (*33*) and sanitation (*34*). These factors, in particular overcrowding, are likely to facilitate transmission of influenza once the disease is present within a community.

Our results suggest that although some communities were severely affected, others may have been less affected by the pandemic because of their isolation. These communities are likely to be particularly susceptible to subsequent waves of infection because East Arnhem communities were particularly hard hit by pandemic (H1N1) 2009 in 2010, and Central Australia communities were relatively spared. Moreover, the first cluster of laboratory-confirmed cases since the first pandemic wave occurred in June and July 2010 in the SLA with the lowest postpandemic proportional immunity of any SLA represented by >20 specimens (2/38, 5.4%).

Our serosurvey indicates that the full effect of the influenza pandemic on the NT may have been underestimated and highlights the differential impact of the virus on vulnerable groups, including children and indigenous populations. Our findings show similarities to other published data, but the results are more likely to be applicable to remote-living and ethnically diverse populations. Given that in all groups, the majority of the population is likely to remain susceptible to the virus following the pandemic, vaccination campaigns and public health responses are essential and should focus on high-risk groups, which requires respectful engagement with communities.
